# Supplementary figures and images for: The Role of the TLR4-MyD88 Signaling Pathway in the Immune Response of the Selected Scallop Strain “Hongmo No. 1” to Heat Stress
Source: Animals (Basel). 2024 Feb 2;14(3):497. doi: 10.3390/ani14030497 (PMC10854496; doi:10.3390/ani14030497)

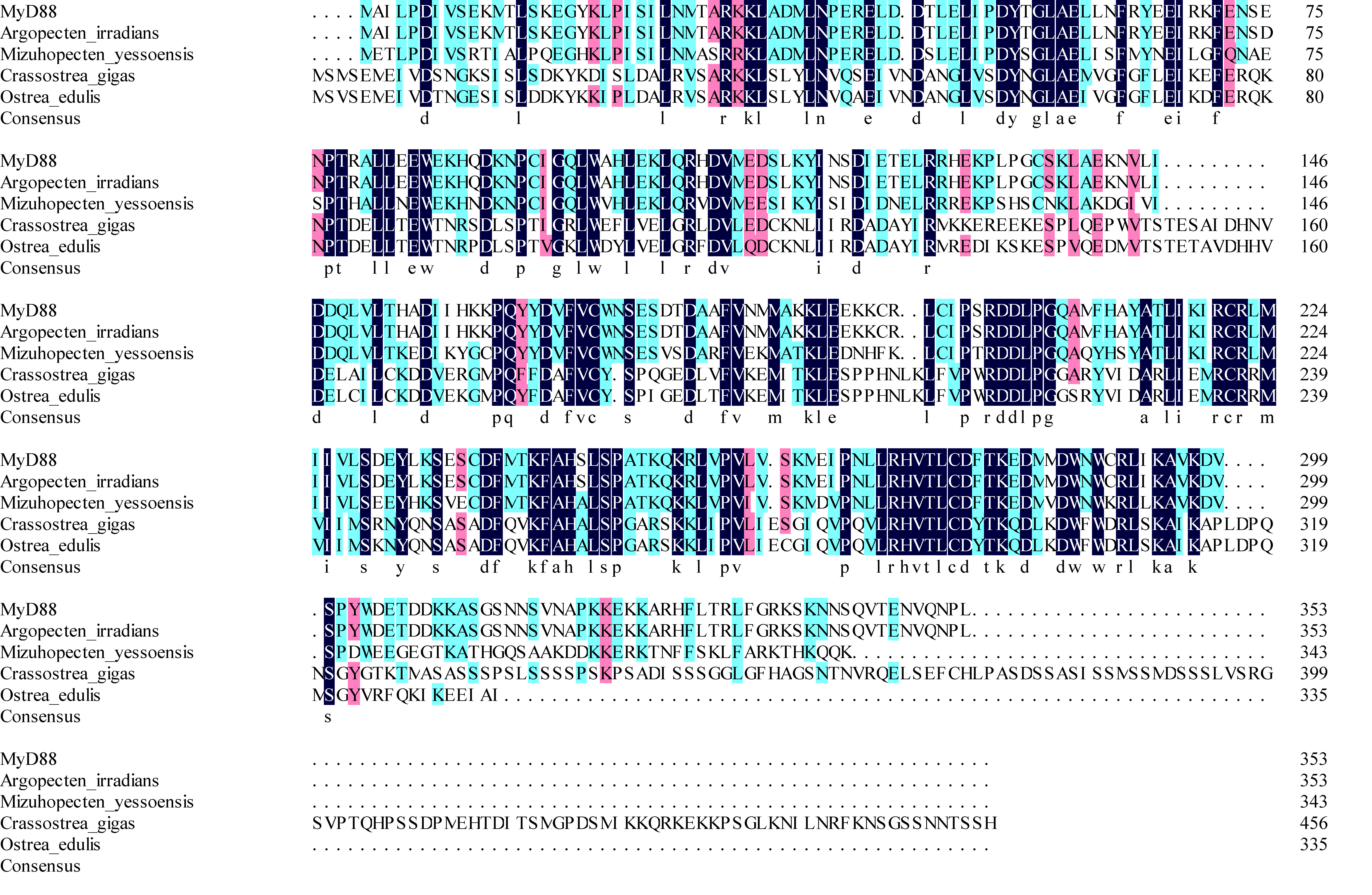

Supplement: Supplementary file 1 [file animals-14-00497-s001.zip › Figure S2.tiff]

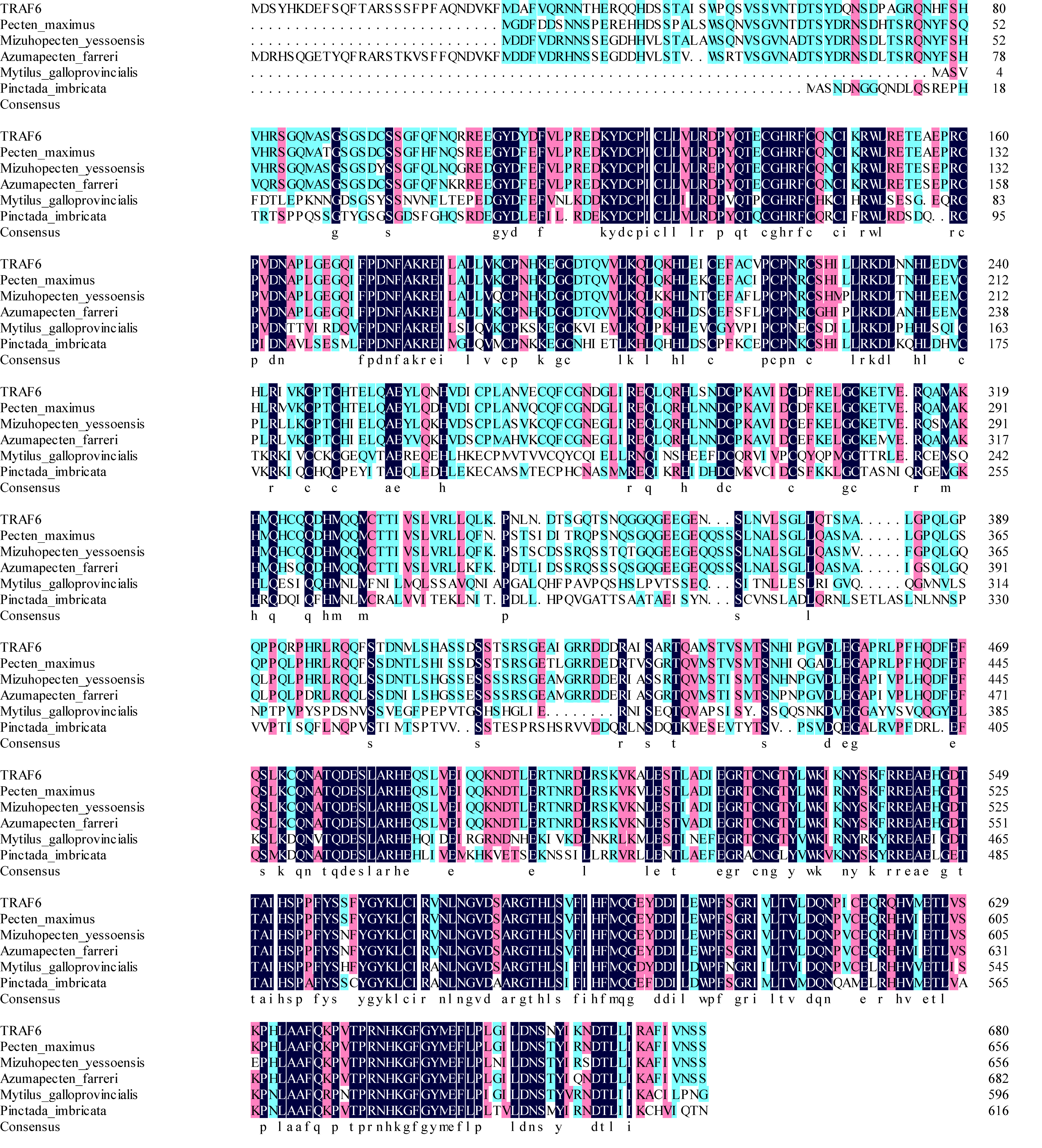

Supplement: Supplementary file 1 [file animals-14-00497-s001.zip › Figure S3.tiff]

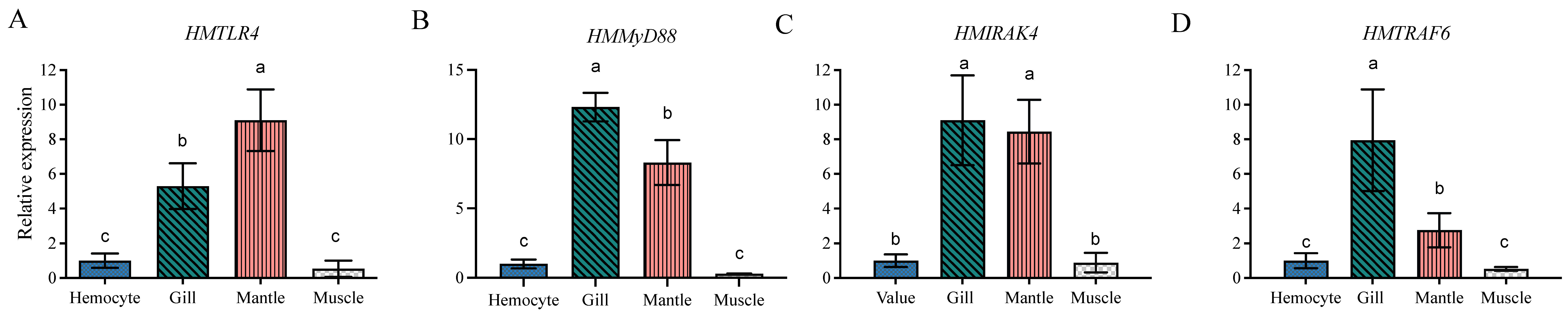

Supplement: Supplementary file 1 [file animals-14-00497-s001.zip › Figure S4.tiff]

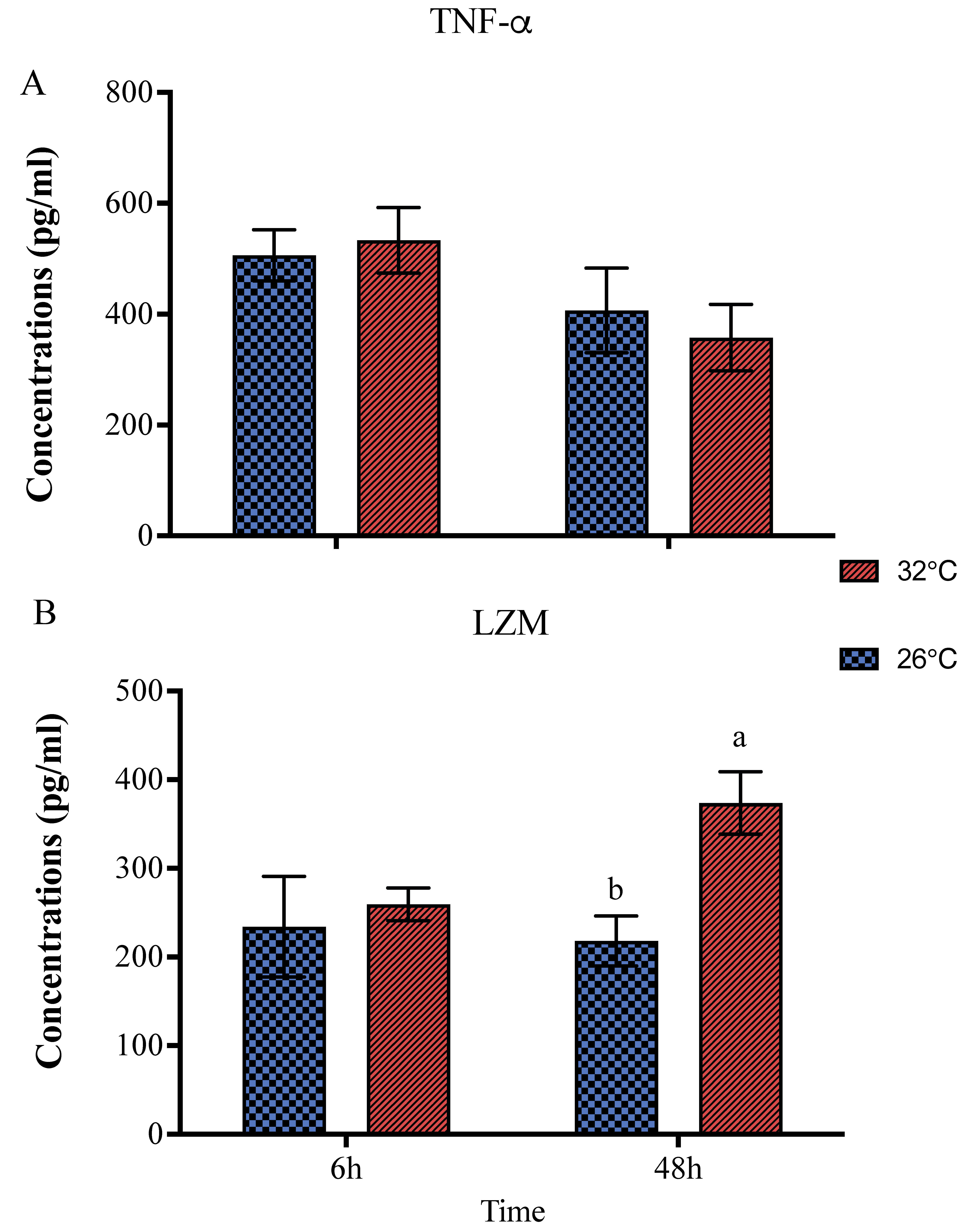

Supplement: Supplementary file 1 [file animals-14-00497-s001.zip › Figure S5.tiff]
